# Experiences of care home staff in the delivery of heart failure care: a grounded theory

**DOI:** 10.1186/s12877-025-06079-1

**Published:** 2025-07-02

**Authors:** Gary Mitchell, James McMahon, Lana Cook, Oonagh McCloy, Paul Tierney, David R Thompson, Laura Creighton, Stephanie Craig, Elizabeth Henderson, Loreena Hill, Jan Cameron, Doris Yu, Debra K. Moser, Karen Spilsbury, Nittaya Srisuk, Jos M G A Schols, Mariëlle van der Velden-Daamen, Christine Brown Wilson

**Affiliations:** 1 School of Nursing and Midwifery, Queen’s University Belfast, Belfast, UK; 2 School of Nursing and Paramedic Science, Ulster Univeristy, Belfast, UK; 3 School of Clinical Sciences at Monash Health, Monash University, Melbourne, Australia; 4 School of Nursing, University of Hong Kong, Hong Kong, China; 5 College of Nursing, University of Kentucky, Lexington, KY USA; 6 School of Healthcare, University of Leeds, Leeds, UK; 7 Faculty of Nursing, Rajamangala University of Technology Thanyaburi, Pathum, Thani Thailand; 8 Department of Health Services Research, Department of Family Medicine, Care and Public Health Research Institute (CAPHRI), Maastricht University, Maastricht, the Netherlands

**Keywords:** Heart failure, Cardiovascular disease, Care homes, Nursing homes, Older people, Gerontology, Grounded theory, Healthcare

## Abstract

**Background:**

Heart failure is a complex syndrome affecting 64 million people globally, with an average patient age of 76 years. Management challenges include medication titration difficulties and patient self-management issues. Care homes, housing approximately 20% of residents with heart failure, face unique challenges in managing this condition. This study aimed to investigate care home staff experiences in supporting residents with heart failure.

**Methods:**

A Glaserian grounded theory approach was employed to explore perceptions, challenges, and strategies used by care home staff in supporting residents with heart failure. Twenty care home staff members from Northern Ireland, with varied roles and experience levels, participated in online semi-structured interviews. These interviews were audio-recorded and transcribed verbatim. Data collection and analysis occurred concurrently, following theoretical sampling principles, between February 2023 and March 2024. A three-stage coding process (open, axial, and selective) was used for analysis. Rigour was ensured through member checking, data source triangulation, and reflexivity. Ethical approval was obtained prior to data collection.

**Results:**

Three main categories were developed from the data: (1) Training, (2) Support, and (3) Communication. Training revealed that care home staff received limited education on heart failure management, primarily focused on acute settings rather than the chronic care needed in care homes. Support highlighted the various facilitators and barriers staff faced in making clinical decisions regarding heart failure care. Communication addressed the experiences of staff in engaging residents and their families about managing heart failure. These categories linked to the core category of (C) Empowerment, which encompassed the challenges staff faced in training, support, and communication. Empowerment illustrated how staff navigated these obstacles to provide effective heart failure care within the unique context of care homes.

**Discussion:**

This study highlights significant challenges in managing heart failure in care homes, including inadequate training, limited professional development, and insufficient support systems. Key barriers include a lack of specialist education tailored to long-term care settings and restricted access to heart failure specialists. Effective communication and proactive care were identified as critical needs, alongside holistic care approaches. Addressing these gaps through targeted education, specialist integration, and evidence-based strategies could empower staff, optimise care quality, and potentially improve outcomes for residents.

**Supplementary Information:**

The online version contains supplementary material available at 10.1186/s12877-025-06079-1.

## Background

Heart failure is a complex, progressive syndrome characterised by structural and/or functional changes to the heart [1–2]. Common symptoms include dyspnoea, fatigue, and oedema, which lead to reduced functional capacity and quality of life [3]. Globally, an estimated 64 million people are living with heart failure, with the average age being 76 years [4–6]. Due to an ageing population, advancements in healthcare, and improved survival rates following cardiovascular events, the prevalence of heart failure is expected to continue rising [7]. Management is primarily pharmacological; however, as many patients experience reduced renal function and/or low blood pressure, medication titration is challenging, often resulting in suboptimal dosing [8]. Non-pharmacological management typically relies on patient self-management, yet this is hindered by limited awareness and understanding of the condition [9]. Consequently, heart failure is associated with high rates of rehospitalisation, morbidity, and mortality. In the United Kingdom (UK), more than 100,000 hospital admissions annually are attributed to heart failure [10].

Following hospital discharge, individuals with heart failure require ongoing management within the community [8]. However, there are notable variations in the quality of care provided to these patients in ambulatory settings across Europe. Factors such as healthcare professional inexperience, co-morbidities, and adverse effects of medications contribute to disparities in pharmacological and implantable cardioverter defibrillator (ICD) management [11]. Education for healthcare professionals and patients, along with multidisciplinary team working, is crucial to optimise care. Research in Sweden revealed that General Practitioners (GPs) often lack sufficient time to provide heart failure education, while nurses require additional training to perform this role effectively [12]. The UK Department of Health has identified multidisciplinary community care as an essential improvement, as 95% of patient care occurs in community settings [13]. In response, initiatives like Acute Care at Home were introduced, allowing individuals with heart failure to receive treatments previously requiring hospitalisation while remaining at home [13]. Patient satisfaction with this service has been high in both the USA and Ireland [14, 15]. However, this approach primarily addresses acute exacerbations rather than the overall management of heart failure. Notably, involving patients in their care and treatment has been shown to reduce hospital admissions and improve survival rates [6].

A significant proportion of community-based care is delivered in care homes, where over 400,000 people reside across approximately 16,000 facilities in the UK [16]. With an average age of 85, it is unsurprising that around 20% of care home residents live with heart failure [17]. Compared to those discharged to their own homes, individuals with heart failure who transition to care homes are often frailer, present with more complex needs, and experience higher rates of rehospitalisation, morbidity, and mortality [18]. Reports indicate that the management and monitoring of heart failure are less robust in care homes than in other community settings [19]. Factors such as inadequate monitoring, insufficient drug prescribing, and suboptimal medication titration contribute to poorer outcomes for care home residents. Care home staff have expressed a desire for greater education on recognising the signs and symptoms of heart failure and identifying exacerbations [20]. Delivering educational interventions could improve staff knowledge, thereby enhancing the quality of care provided to residents [21]. However, sustaining such interventions and quality improvement initiatives in care homes remains challenging [22–24]. Barriers include high staff turnover, which directly impacts the implementation and long-term effectiveness of heart failure guidelines [25–28]. Enhancing the integration, acceptability, and sustainability of interventions is therefore critical [21].

One potential avenue to improve sustainability is through digital health. However, before effective interventions can be designed and implemented, it is necessary to identify and understand the facilitators and barriers to delivering care for care home residents with heart failure. Despite the increasing prevalence of heart failure and the unique challenges of managing this condition in care homes, there is a notable lack of research focusing on the experiences of care home staff in supporting these residents. Care home staff play a crucial role in the daily management of heart failure, but their specific perspectives and challenges remain poorly understood.

To address this gap, this study used a grounded theory approach to investigate the experiences of care home staff in delivering care to older residents (aged 65 and over) with heart failure. By examining staff perceptions, challenges, and strategies, alongside identifying key facilitators and barriers to care, the study seeks to provide an evidence base for improving care practices and informing the development of effective, sustainable interventions. As this study is not a clinical trial, it has not been formally registered.

## Methods

### Aim

The aim of this study was to investigate the experiences of care home staff in supporting residents living with heart failure. Specifically, the objectives were to:

1. Explore the perceptions, challenges, and strategies used by care home staff in supporting people with heart failure.
2. Identify and understand the facilitators and barriers to delivering care for residents with heart failure in care home settings.
3. Develop a theory grounded in empirical data related to supporting residents with heart failure in care homes.

### Design

A Glaserian grounded theory approach was employed for both data collection and analysis [29]. This methodology was chosen due to the limited research available in the field, as it facilitates the development of theory grounded in empirical data, specifically related to supporting residents with heart failure in care homes. Grounded theory ensures that findings are developed directly from the data, increasing their relevance for understanding the current phenomenon and providing a foundation for future interventions [29–32]. The Glaserian approach also emphasises theoretical sensitivity, allowing the research team to recognise patterns of behaviour in the data [33]. This approach offered practical advantages, allowing the research team to maintain a wide-ranging focus while investigating care home staff experiences in delivering and supporting residents with heart failure care [34].

### Sample

The recruitment and data collection process followed a Glaserian grounded theory approach, employing theoretical sampling [35]. This method involved concurrent data collection and analysis, with participant selection based on emerging conceptual needs [35–37]. As the study progressed, each new participant was identified to further explore the research question, guided by developing theoretical categories. The sampling strategy recruited care home staff with varied levels of clinical expertise, years of professional experience, and involvement in leadership roles. This approach enabled a comprehensive exploration of diverse roles within the care home setting in Northern Ireland. The sampling continued until theoretical saturation was reached, ensuring no new patterns or categories emerged from the data [35]. Table 1 provides an overview of all study participants. All participant names have been replaced with a pseudonym to maintain confidentiality.

**Table 1:** Participant characteristics

Pseudonym	Staff Role	Gender	Experience (Years)
Abigail	Home Manager	Female	1–5 years
Benjamin	Care Home Nurse	Male	1–5 years
Charlotte	Home Manager	Female	5–10 years
Daniel	Care Assistant	Male	0–1 year
Eleanor	Activity Coordinator	Female	10 + years
Francesca	Care Home Nurse	Female	1–5 years
Gabriel	Kitchen Assistant	Male	1–5 years
Hannah	Charge Nurse	Female	1–5 years
Isabella	Home Manager	Female	1–5 years
James	Activity Coordinator	Male	1–5 years
Katherine	Home Manager	Female	10 + years
Liam	Care Home Nurse	Male	1–5 years
Margaret	Charge Nurse	Female	1–5 years
Natalie	Care Assistant	Female	1–5 years
Olivia	Care Assistant	Female	0–1 year
Penelope	Home Manager	Female	1–5 years
Quentin	Home Manager	Female	1–5 years
Rebecca	Care Home Nurse	Female	5–10 years
Sophia	Charge Nurse	Female	1–5 years
Theresa	Care Home Nurse	Female	1–5 years

### Recruitment

Study participants were recruited via the Royal College of Nursing’s Independent Sector Network in Northern Ireland by email through a gatekeeper not involved in the study. This network includes care home staff such as home managers, charge nurses, and nurses. Those interested in participating contacted the research team directly after receiving the gatekeeper’s email to arrange an interview. To achieve theoretical saturation, we also sampled care assistants who were registered with the Northern Ireland Social Care Council (NISCC) (https://niscc.info/). All nurses were registered with the Nursing and Midwifery Council (NMC) (https://www.nmc.org.uk/). As part of the theoretical sampling process, one kitchen assistant registered with NISCC was included in the study. This decision occurred during the analysis as the importance of meal preparation, salt intake and fluid intake for residents with heart failure became apparent. Care assistants and the kitchen assistant were not part of the initial recruitment network and represented a harder-to-reach population. Therefore, snowball sampling was employed to facilitate their inclusion in the study. Interested participants contacted the research team directly to receive further information and arrange interviews. Written consent was obtained via an online form at least 48 h before the scheduled interview and reconfirmed immediately prior to the interview. The diverse sample of participants, including those from various roles and responsibilities within the care home setting, was an important way to capture a detailed understanding of heart failure care experiences and perspectives from within care homes.

### Data collection

Twenty care home staff participated in one-to-one semi-structured interviews with one member of the research team (GM). All participants chose to be interviewed online using Microsoft Teams. Seventeen care homes were represented in total, as three pairs of participants – Isabella and James, Charlotte and Eleanor, and Abigail and Gabriel came from the same care homes. These overlaps occurred due to the challenges of recruiting for non-direct care roles during theoretical sampling. Initially, the recruitment strategy targeted only direct care staff, and the research team relied on care home managers, interviewed earlier in the study, to help facilitate contact with staff in activity coordination and kitchen roles. All participants chose to be interviewed online using Microsoft Teams. The interview guide was developed with input from the authors and the steering group of the Royal College of Nursing’s Older People’s Forum (https://www.rcn.org.uk/Get-Involved/Forums/Older-Peoples-Forum). This ensured that the questions were relevant, comprehensive, and aligned with the study’s objectives while addressing key issues in the delivery and support of heart failure within care home settings. The semi-structured interview guide was also refined and developed throughout the study by the process of theoretical sampling [35–37]. This resulted in the modification and addition of questions to the guide as interviews with participants progressed, which is a key component of grounded theory methodology. For example, the question *“How do you implement and manage exercise-based rehabilitation activities for residents with heart failure?”* was added after the fourth interview to explore the emerging theme of integrating physical activity into heart failure management in care homes. The interview guide can be viewed in supplementary file 1. Data were collected between February 2023 and March 2024.

### Ethical considerations

#### Ethical approval

for this study was granted by the Faculty of Health and Life Sciences at Queen’s University Belfast in October 2022 (Ref: MHLS22_112). Written informed consent was obtained from all twenty care home staff who participated. The study adhered to the Declaration of Helsinki, participant privacy and confidentiality was protected throughout. During data anonymisation, all personal identifiers were removed to protect participants’ identities. Interview transcripts and other data were securely stored with access restricted to the research team. Direct quotations included in the study were anonymised to prevent identification of individual participants or patients. These protocols were followed to uphold ethical standards and safeguard the confidentiality of all participants.

### Data analysis

The analysis adhered to the principles of constant comparative analysis, a key aspect of Glaserian grounded theory methodology, involving simultaneous data collection and analysis [29, 34, 37]. Each interview was examined before the next, allowing analysis to shape subsequent data collection. After verbatim transcription, a three-stage coding process was conducted: open coding to identify key concepts, axial coding to explore relationships between categories, and selective coding to determine a core category connecting all others [34]. This approach supported understanding of the data, refinement of themes and development of concepts. Primary analysis was conducted by two experienced researchers, with input from the wider research team to ensure rigor.

### Rigour

The research team implemented several strategies to enhance the credibility and trustworthiness of the study’s findings, adhering to the Consolidated Criteria for Reporting Qualitative Research (COREQ) guidelines [38]. As categories were developed from the data, they were shared with three study participants for validation, ensuring accurate reflection of their experiences and the applicability of the emerging theory. This process, known as ‘member checking’, provided assurance that the theory was firmly grounded in the data. In line with theoretical sampling principles, the team conducted data source triangulation by interviewing care home staff of varying roles, experience levels, and backgrounds. This approach, while potentially impacting traditional data saturation, served to enrich the qualitative analysis by capturing a range of perspectives on heart failure care in care home settings. Further, rigour was also maintained through regular team meetings and active involvement of all research team members, as evidenced by a detailed audit trail. Reflexivity was practiced, with researchers acknowledging and discussing their own positions and potential biases in relation to the research topic. Members of the research team directly involved in data collection and analysis also engaged in peer debriefing, where emerging findings and interpretations were discussed with colleagues not directly involved in the study, providing alternative perspectives and considering conflicting assumptions.

## Results

Three categories were developed from the data to support the development of the core phenomenon. These three categories were: (1) Training, (2) Support and (3) Communication. The core category in this study, which was apparent across all the categories, was defined as (C) Empowerment. The core category ‘empowerment’ encompassed all other categories and was developed to explain how participants in this study navigated (1) training gaps that limited their education on heart failure management and symptom recognition, (2) the availability and adequacy of supportive structures, including access to clinical expertise, the use of tailored care plans, and the provision of relevant resources, which influenced the extent to which staff were able to make informed clinical decisions, and (3) communication experiences in engaging with residents, families and professionals to foster a shared understanding of heart failure care.

### Category 1: training

The first category to be developed from the data related to care home staff education about heart failure. Participants reported limited exposure to heart failure education during their pre-registration nursing programmes. While training provided a foundational understanding of heart failure management, it was primarily geared toward acute hospital or rehabilitation settings rather than chronic care scenarios within care home settings. This misalignment in education left care home staff feeling unprepared to address the complexities of heart failure in this unique context.

“I remember learning about heart failure during training, but it was all hospital-based scenarios. We never talked about how to manage these patients in a care home, where the challenges are a bit different” [Francesca, Care Home Nurse].

Participants emphasised that while hospital and rehabilitation settings provide controlled environments with a multidisciplinary team approach, the same level of resources is not available in care homes. As a result, the knowledge acquired during pre-registration education often fell short of addressing the chronic and multifaceted needs of nursing home residents with heart failure.

“The training was good at teaching us about things like medication titration and managing acute exacerbations in hospital settings. But it didn’t teach us how to manage someone with heart failure and dementia or someone who is entirely dependent” [Margaret, Charge Nurse].

Another gap identified was the lack of bespoke training programmes tailored to the unique challenges of managing heart failure in residents with significant comorbidities and varying levels of dependency. Participants also stated that managing heart failure in care homes is complex because it requires a detailed understanding of how heart failure interacts with other long-term health conditions like frailty or dementia.

“We’ve got people who have heart failure and five or six other conditions. They might be bedbound or have cognitive issues too. The training we had didn’t equip us to deal with this level of complexity—it’s completely different from managing heart failure in a younger, more independent patient who can comply with rehabilitative care” [Katherine, Home Manager].

The absence of ongoing clinical education specific to heart failure in the care home context further compounded these challenges. Participants pointed out that most continuing professional development (CPD) opportunities usually focused on generalised care of older people or dementia, with little to no emphasis on heart failure management.

“I’ve done lots of CPD since qualifying [as a registered nurse], but I can’t think of a single session that focused on heart failure. We get general updates on managing frailty or end-of-life care, but nothing about heart failure…nothing at all” [Liam, Care Home Nurse].

This lack of ongoing education often left care home staff reliant on their initial training or ad-hoc resources, which they felt were insufficient to keep pace with advances in heart failure management. Many participants expressed concern that without tailored education, they might not be providing the most up-to-date and effective care for their residents.

“I think we’re all probably doing our best with the knowledge we have, but heart failure is complex, and there are always new treatments or guidelines. It would be good to have access to proper training so we can be confident we’re doing the right thing” [Quentin, Home Manager].

In addition to gaps in nursing education, the study revealed a striking lack of knowledge about heart failure among care assistants. Most care assistants participating in the study admitted they were unsure what heart failure was. Several believed it referred to a person dying, with one participant associating the term with their CPR training and the process of resuscitation. This paucity of education highlighted the limited understanding of heart failure among care assistants, who play an important role in the care of residents. Care assistants unanimously agreed that they needed more education about heart failure. They emphasised that as the primary caregivers, they are often best placed to be the first professional to notice changes in a resident’s condition. For example, care assistants are responsible for helping residents reposition in bed, a task that can reveal fluid retention in the feet. They also provide fluids to residents and spend the most time with them, making them well-placed to observe signs of fatigue or breathlessness which are potential indicators of worsening heart failure.

“We’re the ones who are with the residents most of the time. If they’re more tired than usual or struggling to breathe, we would notice first. But if we don’t know what heart failure looks like, how can we help?” [Natalie, Care Assistant].

Other staff members, including activity coordinators and kitchen staff, also demonstrated a lack of awareness about heart failure and its management. Activity coordinators, who could potentially support rehabilitative exercises for residents with heart failure, were unaware of which residents had the condition. Similarly, kitchen staff, who are responsible for meal preparation, lacked knowledge about dietary considerations for heart failure. A kitchen assistant expressed concern about their limited understanding, especially regarding specific dietary needs:

“I wouldn’t know if someone had heart failure, and I’ve never been told what kind of food they should have or avoid. I just follow the general menu plan, but maybe they need something special that I don’t know about.” [Gabriel, Kitchen Assistant].

An activity coordinator also highlighted how being informed about residents with heart failure would allow them to better tailor activities to support mobility and well-being:

“If I knew who had heart failure, I could tailor exercises or activities to help them stay mobile. But honestly, I’ve never been given that information, so I wouldn’t know where to start.” [James, Activity Coordinator].

Overall, this theme revealed significant gaps in heart failure education among care home staff, with pre-registration training often focused on hospital-based care rather than chronic management within long-term care facilities. Ongoing clinical education was also lacking, leaving staff reliant on outdated knowledge. Care assistants, who spend a lot of time with residents, lacked awareness of heart failure symptoms and were unable to act on early signs of deterioration. Additionally, non-nursing staff, such as activity coordinators and kitchen assistants, were unaware of how to support residents with heart failure, highlighting the need for comprehensive, tailored education across all roles.

### Category 2: support

A significant theme that was developed from the data pertained to the support that care home staff received in managing residents with heart failure. Participants expressed several challenges in providing optimal care to individuals with heart failure due to inadequate support systems, lack of specialised training, and limited access to resources and evidence-based guidelines.

First, access to General Practitioner (GP) support was a recurring issue across the care home settings. Participants described difficulty in obtaining routine monitoring and support from GPs, noting that visits were often limited to acute situations rather than ongoing care. This lack of regular GP involvement left care home staff with limited guidance on managing the day-to-day needs of residents with heart failure. One care home nurse described the situation as follows:

“GPs only come when things are really bad, like when the patient is really struggling. But heart failure doesn’t just flare up suddenly, so we don’t get the support we need for regular reviews or monitoring” [Rebecca, Care Home Nurse].

In addition to the lack of consistent GP support, the use of generic care plans, such as those based on Roper, Logan and Tierney’s [39] Activities of Daily Living (ADLs), was seen as a major limitation. These care plan templates did not address the specific and complex needs of heart failure management. The participants noted that the generic nature of these plans failed to incorporate critical aspects of heart failure care, such as monitoring fluid balance, medication titration, or managing acute exacerbations. A care home manager emphasised this gap:

“Our care plans are based on general activities like dressing, eating, and bathing, but they wouldn’t be very inclusive of people with heart failure. So, when the girls [registered nurses] are doing their assessments and care plans based on our bundle [care home documentation], they might not pick up residents who are feeling more sleepy or residents with more fluid accumulation or those with a bit more breathlessness…certainly something a bit more tailored [to heart failure assessment] would help us” [Penelope, Home Manager].

Another significant barrier identified was the apparent absence of community-based heart failure specialist nurses or cardiologists to guide and support care home staff. Many participants were unaware of how to access these specialised professionals or lacked knowledge about what resources were available to them. This lack of specialist input left care home staff feeling isolated and ill-equipped to address the complexities of heart failure management. A nurse described the situation as follows:

“We don’t have heart failure nurses coming to support us, and we don’t even know how to get in touch with them…or even if they exist out there! We’re really left to manage on our own, and it sure can be overwhelming” [Benjamin, Care Home Nurse].

Furthermore, the lack of resources and programmes focused on heart failure prevention within care homes was highlighted as a major challenge. Care home staff expressed frustration over the absence of materials, research journals, or even access to computers to stay informed on the latest guidelines and evidence on heart failure care. One participant mentioned:

“We don’t have access to journals or computers to research heart failure care. If we don’t have the evidence, how are we supposed to provide the best care? It feels like we’re just going with what we know, without any guidance” [Sophia, Charge Nurse].

Despite these barriers, there were a few positive aspects of care in the homes. Some participants noted that palliative care services were sometimes involved with residents who had heart failure, though this typically only occurred in end-of-life scenarios or once symptoms had become severe. This awareness of the palliative nature of heart failure was seen as an important facilitator, though it was often applied reactively rather than proactively. One care home manager reflected:

“Heart failure is often recognised as a palliative condition, but it’s usually when things are getting really bad. It would be better if we were involved earlier on to help manage symptoms before they become so severe” [Katherine, Home Manager].

Similarly, the use of advance care plans, although not widespread, had proved beneficial in some instances, particularly in managing acute exacerbations and avoiding unnecessary hospital admissions. Participants highlighted that when advance care plans were in place, staff could provide more tailored care and respect residents’ wishes. One nurse commented:

“Advance care plans have really helped with managing people with heart failure, especially when they have exacerbations. It’s been helpful in preventing unnecessary trips to the hospital and keeping people more comfortable” [Margaret, Charge Nurse].

Additionally, staff with prior experience in palliative care appeared to bring valuable knowledge into their practice, albeit usually in an informal, incidental way. This knowledge helped to manage heart failure in some cases, but there was often no formalised system for disseminating or building on this expertise across the care home setting. One care home worker with a background in palliative care explained:

“I used to work in palliative care, so I know how to manage symptoms like breathlessness or fluid retention. But I don’t think anyone else in the team has had this training. It’s just something I do because of my past experience in hospice care” [Hannah, Charge Nurse].

Another issue highlighted was the high turnover of staff and reliance on agency workers, which contributed to a lack of continuity in care. Staff who were not familiar with the residents were less likely to notice subtle changes in their condition, such as fluid retention or increased fatigue, which could signal a worsening of heart failure. One participant observed:

“Agency workers come in and don’t really know the residents well. If they don’t have a relationship with the patient, they might miss the early signs of heart failure worsening” [Olivia, Care Assistant].

Despite these challenges, participants noted that care home staff did engage in public health initiatives like promoting healthy eating and encouraging physical activity, which could also benefit residents with heart failure. However, the lack of awareness among staff about the link between these behaviours and heart failure management meant that such efforts were often not targeted at preventing or managing heart failure specifically as noted below.

“We do encourage healthy eating and exercise, but I didn’t really think about how this could help prevent heart failure. I suppose it does. We just sort of do it…because it is good for them [the residents]…you know?” [James, Activity Coordinator].

“Of course, healthy meal choices, regular fruit and veg (vegetables), low salt, low sugar – you name it, we do it. It’s not specific to heart failure, I wouldn’t know each person’s medical condition or anything like that, but absolutely, every effort is made to promote healthy eating” [Gabriel, Kitchen Assistant].

In this theme it was evident that care home staff faced significant challenges in providing adequate care for residents with heart failure due to a lack of adequate support from healthcare professionals and limited access to resources. While some positive practices, such as the use of advance care plans and the involvement of palliative care services, were observed, it is apparent that there is more that needs to be done to equip staff with the support necessary to manage heart failure proactively.

### Category 3: communication

The third category developed from the data highlights the role of communication processes in the care of residents living with heart failure. Participants consistently described challenges and barriers related to communication, both within the care home and with external professionals and families. Despite some examples of effective communication, it was evident that poor communication practices significantly impacted the quality of heart failure prevention, assessment and management.

One key issue was uncertainty about whom to contact for external support regarding heart failure management and when to do so. Participants described confusion about whether to involve GPs, cardiologists, hospital services, ambulance services, palliative care teams, mental health professionals, or physiotherapists. This uncertainty often led to reactive communication during crises or exacerbations rather than proactive, planned coordination as noted in the three excerpts that follow:

“We don’t know all of the people to contact, but if something goes wrong, we would need to get the resident admitted [to a hospital].” [Charlotte, Home Manager].

“To be honest, I’d call a GP if I was concerned…maybe an ambulance. If they [the resident] are deteriorating there is not much we can do [at the care home]” [Theresa, Care Home Nurse].

“In an ideal world, sure, there are lots of guys and girls [multidisciplinary professionals] that could do a whole lot. But everything is in crisis [health service]. Budget cuts, staff cuts…everything is shite. The only people they [the resident] can rely upon is us [care home staff]” [Daniel, Care Assistant].

This reactive approach was further compounded by the difficulty in accessing timely GP support, as noted in the first category. Without clear guidance or protocols, care home staff often felt unsupported in managing heart failure effectively. Poor communication with families, particularly regarding advance care planning and palliative care options, was another significant challenge. While ACP was noted as effective when implemented, it was inconsistently applied, particularly for residents with HF. Care home staff highlighted that ACP discussions were more common for conditions like cancer or dementia, whereas HF was often perceived as a “silent” or “invisible” illness when well-managed. One participant explained:

“Okay, so families don’t always know heart failure is incurable or palliative, right? So, when the resident seems stable, no one wants to talk about it. Not the family, not the staff. It’s like we’re avoiding the issue.” [Charlotte, Home Manager].

This lack of awareness and communication sometimes led to missed opportunities for families to prepare for the progression of heart failure and for residents to express their preferences for future care. Communication challenges also extended to discussions with residents themselves about prognosis and future care planning. Staff acknowledged a tendency within care homes to “do for” residents rather than to promote independence, which could be counterproductive for those with heart failure. Examples of proactive care, such as armchair aerobics or encouraging short walks with support, were highlighted as beneficial but not consistently applied. The fragmented communication among staff, who often worked in shifts and varied roles, made it difficult to reinforce these positive practices. A care home worker remarked:

“We try to encourage independence, but with so many staff coming and going, it’s hard to keep everyone on the same page. When we are down [short-staffed], we don’t really get or take the time to promote independence” [Hannah, Charge Nurse].

Limited interdisciplinary communication or integration further compounded the issues in managing complex heart failure cases. Nurses in this study often perceived heart failure care as the responsibility of external specialists, while care assistants felt it should be led by the nurses in the care home. This lack of clarity about roles and responsibilities created gaps in care. Additionally, care assistants reported that they were seldom informed about which residents had heart failure or how best to support their individualised care. One care assistant shared:

“We don’t get told much about medical conditions like heart failure. We just focus on the daily tasks, so yeah, I think we could miss things that might be important.” [Natalie, Care Assistant].

Nurses in this study conceded that at times their communication with care assistants often prioritised social care tasks over medical needs. Daily handovers and reports led by the nurses before the beginning of a shift was said to rarely include medical histories, information about ACPs, or specific guidance on heart failure management for people with the condition. Instead, discussions were usually focused on topics like nutritional intake, mood, medications and wound care. A senior nurse noted:

“We tend to focus on what’s immediately relevant to the day, like food and mood. Heart failure management doesn’t usually come up unless there’s a specific problem.” [Margaret, Charge Nurse].

This disconnection extended beyond care assistants to other support staff, such as activity coordinators and kitchen assistants, who received little to no information about residents with heart failure. While these roles were less central to direct care, their involvement could potentially contribute to holistic management if they were better informed. Despite these challenges, there were some positive examples of communication and collaboration. When ACPs were in place, they were described as effective in helping residents and families prepare for the progression of heart failure and in avoiding unnecessary hospital admissions. Participants also noted that proactive communication with palliative care services could improve symptom management during end-of-life care.

“In cases where we’ve used advance care plans, it’s been easier to manage exacerbations. The resident is more comfortable, and the family feels reassured.” [Isabella, Home Manager].

Additionally, interdisciplinary approaches within care home settings could show promise in fostering better communication, but these were not yet widespread. Some care homes were exploring ways to improve integration, such as introducing team meetings that included both nursing and support staff. While not specific to heart failure care, such an approach could be beneficial as highlighted by one home manager:

“I think if we could bring everyone together regularly—nurses, care assistants, domestics, activity therapists, it would help us work as a team and focus more on individual needs. We’d be stronger together. That’s my plan [Quentin, Home Manager].

Across this third category, it was evident that communication processes play an important role in the prevention, assessment and management of heart failure. While there were examples of effective communication, such as the use of ACPs and collaboration with palliative care, significant gaps remain. Challenges include uncertainty about contacting external support, poor communication with families and residents, fragmented interdisciplinary communication, and inconsistent sharing of information among staff.

### Core category: empowerment

In grounded theory methodologies, identifying a core category is integral to unifying the data and building theory [29, 35]. The core category serves as a central concept, incorporating all aspects of the study, and is developed through analysis of the dataset [34, 35]. In this study, the core category identified was empowerment. This concept was developed as it recurred across all facets of the research and directly interacted with the identified categories of (1) training, (2) support, and (3) communication. Empowerment provided a cohesive lens through which to understand the participants’ experiences and challenges in heart failure management in care home settings.

Considering (1) training, the participants frequently highlighted gaps in their training related to heart failure management and symptom recognition. This lack of training created barriers to feeling empowered in their roles. Many participants reported they were not confident in their ability to recognise subtle signs of heart failure exacerbation. Moreover, limited opportunities for continued professional development compounded this challenge. One participant explained.

“We are expected to be experts in everything, but the truth is, I don’t remember if we have ever [the care home staff] received any training on heart failure. It’s a total blind spot.” [Penelope, Home Manager].

This uncertainty in training is likely to have created a ripple effect, influencing participants’ confidence in clinical decision-making and resident care. Despite these challenges, participants expressed a desire to bridge these gaps, emphasising the need for structured, ongoing education to improve their competence and autonomy. Further, the role of (2) support in developing empowerment was another key finding. Participants described a dichotomy between facilitators of support—such as mentorship from experienced colleagues—and limitations, including time constraints and understaffing, which impeded their decision-making processes. Support, or the lack thereof, appeared to directly impact their ability to navigate complex clinical scenarios with confidence. One participant noted:

“When I have someone to turn to, like a good GP or my clinical sister, I feel like I can manage even the most difficult situations. But when I’m on my own or it’s a weekend shift, it’s a different story. You feel unsupported, and that impacts the care you can give to your resident.” [Liam, Care Home Nurse].

The lack of internal support within the care home setting was also highlighted, with many participants describing challenges in accessing resources or systemic guidance on managing heart failure in their care setting. This variability in support led to further feelings of disempowerment, highlighting the need for openly accessible evidence to assist nurses in their roles.

The third category of (3) communication also played a pivotal role as either a facilitator or barrier to the process of staff empowerment. Participants shared that engaging with residents and families to create a shared understanding of heart failure care was often fraught with challenges. Miscommunication or differing expectations could lead to conflict, which participants found disempowering. For example, one participant recounted,

“Sometimes families don’t understand what heart failure means. Their expectations are perhaps different than yours would be as a nurse. It’s hard to explain it [heart failure] is an incurable condition that will probably get a lot worse without upsetting them, and you end up questioning yourself.” [Sophia, Charge Nurse].

However, positive communication experiences, where mutual understanding about heart failure was achieved, served as a source of empowerment. Nurses who were able to effectively convey complex medical information and involve families in decision-making felt more confident and capable in their roles.

Across all three categories, (1) training, (2) support, and (3) communication, the concept of empowerment was central. Participants’ experiences illustrated that empowerment was not a static state but rather a dynamic process influenced by their educational opportunities, the support structures available to them, and their ability to communicate effectively with multidisciplinary professionals, residents and families. Empowerment, or the lack thereof, was therefore pivotal in shaping how care home staff perceived their roles and navigated challenges in heart failure care. One participant eloquently summarised the importance of empowerment, stating:

“It’s not just about knowing what to do, it’s about feeling like you can do it, that you have the backing of your team, the right training, and the ability to make a difference to the resident. That’s what makes you feel able to make a difference and that’s why we do the damn job!” [Francesca, Care Home Nurse].

## Discussion

The findings of this study highlight significant challenges in managing heart failure in care homes. Training deficiencies were consistently identified as a significant barrier to effective heart failure care in care homes. Participants highlighted the inadequacy of pre-registration nursing education, which often prioritises acute and hospital-based care settings over the unique needs of long-term care residents. Specific training on recognising and managing early symptoms of heart failure exacerbation was notably absent, leaving staff feeling unprepared to address the complexities of care and these findings have been echoed within the international literature [40, 41]. Furthermore, continuing professional development opportunities tailored to heart failure management in nursing homes were limited, a gap that has been shown to negatively impact the confidence and competency of staff [40, 42, 43]. Addressing these training gaps with targeted, context-specific education is essential for empowering care home staff and improving resident outcomes.

The absence of adequate support systems further compounds the challenges in managing heart failure. Access to heart failure specialist nurses, who can provide guidance and evidence-based strategies, was notably lacking in this study. Such roles have proven instrumental in improving care quality in community and hospital settings and should be integrated into long-term care environments. A Cochrane review found that specialist nurse-led care for heart failure patients was associated with statistically significant reductions in all-cause hospital admissions and decreases in heart failure-related admissions compared to usual care, demonstrating their significant impact on patient outcomes [44]. Similarly, a seminal study conducted more than twenty years ago, focusing on heart failure nurses in primary care settings found that patients under their close monitoring were less likely to be readmitted to hospital, and those who were admitted often experienced shorter hospital stays, emphasising the cost-effectiveness of these roles two decades earlier [45]. Recent research provides substantial evidence indicating that healthcare professionals with specialist expertise in heart failure consistently achieve improved patient outcomes compared to those without specialised training [46, 47]. International studies have demonstrated that heart failure specialist nurses, for example, are instrumental in reducing hospital readmissions, lowering mortality rates, and enhancing patients’ quality of life [48, 49]. These specialists bring focused knowledge and skills that enable more precise symptom management, timely interventions, and effective patient education [46–49]. Moreover, they play a pivotal role in coordinating care across multidisciplinary teams, ensuring continuity and reducing fragmentation of care, which is particularly critical in managing complex conditions like heart failure. The integration of heart failure specialist practitioners to support care homes has the potential to address these gaps, providing the necessary expertise to manage the unique challenges within this setting. Their presence could empower care home staff to deliver higher-quality, evidence-based care while reducing the reliance on hospital services for residents with heart failure.

In this study, effective communication has been identified as an important aspect of care for residents with heart failure, yet it remains a significant challenge. Participants expressed uncertainty about when and how to engage external professionals or escalate concerns, a finding echoed in similar studies [50, 51]. Poor communication between care home staff, residents, family and healthcare providers can delay interventions and increase the risk of hospitalisations [52]. Similarly, communication with residents and their families regarding prognosis and care goals was also often found to be inadequate in this study. Advance care planning, an essential component of heart failure management, appeared to show evidence of promise for residents with heart failure in care homes but was limited by staff discomfort and lack of training in initiating these sensitive discussions and this is consistent with the literature about advance care planning in care homes [53, 54] [55]. Clearer communication pathways and targeted training have demonstrated increased effectiveness in advance care planning and positive resident outcomes [56, 57]. While care home staff showed a willingness to engage with external healthcare professionals, the data revealed a lack of formal training or guidance on effective team-based communication. Interactions were often informal and inconsistent, with staff relying on personal judgment rather than structured approaches. Notably, there was no reference to the use of recognised interprofessional communication frameworks such as SBAR (Situation, Background, Assessment, Recommendation) or ISBAR, which have been shown to improve information transfer, reduce communication errors, and support clearer clinical decision-making across settings [58–60]. The absence of such structured tools may have contributed to delays in escalation, fragmented care, and unclear role boundaries. Incorporating standardised handover models into routine care home practice could improve continuity of care and collaborative decision-making, particularly for residents with complex conditions such as heart failure, by ensuring timely symptom recognition, consistent documentation, and more coordinated care planning.

Organisational culture and hierarchy appeared to influence communication and collaboration in care homes. Many participants suggested that communication often flowed in a top-down manner, with nurses potentially acting as gatekeepers to external clinical teams. Care assistants, kitchen staff, and activity coordinators, despite frequent contact with residents, seemed to be less involved in medical discussions. This hierarchical structure may have limited opportunities for holistic, team-based care. Participants expressed frustration about their potential inability to access specialist services, which may point to disempowerment in interactions with external healthcare systems. Addressing these barriers could be important for fostering more collaborative, person-centred care for residents with complex conditions such as heart failure [61–63].

A proactive approach to heart failure care is crucial for preventing disease progression and avoiding hospital admissions. Early identification of at-risk individuals through better assessment tools and monitoring can significantly reduce the incidence of heart failure in older adults [64]. For residents already diagnosed with heart failure, routine monitoring and early intervention can help manage exacerbations and improve outcomes [65]. Preventive measures, including lifestyle modifications such as dietary changes and exercise programmes, should be integrated into care plans to reduce risk factors and promote cardiovascular health [66]. Developing tailored assessment tools and protocols for early intervention is essential for optimising care and reducing the burden of heart failure in care homes was also found to be important within this study.

Despite the complexity of heart failure, the study findings suggest a lack of holistic consideration in its management. Participants did not routinely emphasise psychological, social, or spiritual aspects of heart failure care, indicating a narrow focus on clinical and physical management during data collection. Holistic care, which addresses the broader needs of residents, is fundamental to improving quality of life and outcomes for individuals with heart failure [67–69]. The integration of palliative care principles into heart failure management is imperative as a means of providing a more comprehensive approach, ensuring that residents’ preferences and goals are respected [70]. Empowering care home staff with targeted education, effective communication strategies, and access to specialist resources appears to be essential to optimising heart failure care for residents. Addressing gaps in training and support systems could enhance staff competence and confidence, enabling timely interventions and reducing hospital admissions. Integrating evidence-based practices and proactive care measures could also ensure residents receive high-quality, comprehensive care tailored to their unique needs.

### Strengths and limitations

This study’s use of Glaserian grounded theory methodology to explore the under-researched experiences of care home staff in supporting residents with heart failure is a strength, allowing for the development of a theory grounded in empirical data. Theoretical sampling ensured diverse perspectives by including staff with varying roles, experience levels, and backgrounds, improving the richness of the findings. Credibility and trustworthiness were maintained through member checking, data source triangulation, regular team meetings, and reflexivity. However, the recruitment method, primarily via the Royal College of Nursing’s Independent Sector Network in Northern Ireland, will have excluded some staff, limiting diversity. Additionally, the use of online semi-structured interviews via Microsoft Teams, while convenient, could have affected rapport and limited insight into non-verbal cues.

## Conclusion

This study highlights significant challenges in managing heart failure in care homes, including gaps in education, support systems, and communication processes. The findings emphasise the need for care home-specific interventions to address these deficiencies. Targeted training programmes, integration of specialist support, and the establishment of clear communication pathways are critical to improving care quality and outcomes for residents. Further research is required to develop and evaluate effective, sustainable strategies to enhance staff competence and optimise heart failure management in care home settings.

## Electronic supplementary material

Below is the link to the electronic supplementary material.

Supplementary Material 1

## Data Availability

The datasets used and/or analysed during the current study are available from the corresponding author on reasonable request.
