# Supplementary material for: Experiences of care home staff in the delivery of heart failure care: a grounded theory
Source: BMC Geriatr. 2025 Jul 2;25:446. doi: 10.1186/s12877-025-06079-1 (PMC12220550; doi:10.1186/s12877-025-06079-1)
Supplement: Supplementary file 1 — Supplementary Material 1 [file 12877_2025_6079_MOESM1_ESM.docx]

**Supplementary File 1: Semi-Structured Interview Guide**

**Interview Protocol**

The interviewer will introduce him/herself and reiterate the purpose of the interview, i.e. to explore nurses’ attitudes towards heart failure healthcare provision for older adults living in care homes, and the barriers they experience providing this type of service. The interviewer will reiterate that the interview will be audio recorded (via MS Teams) and transcribed for research purposes, but transcripts will be anonymised i.e. will not contain participant names. In addition, it will be made clear to participants that they can inform the facilitator if there are any statements they do not wish to be transcribed at the end of the session. The interviewer will explain to the participants that they are free to stop the interview at any stage if they feel uncomfortable with any of the discussions or do not wish to participate further.

The interviewer will ask if participants have any questions before starting the discussion.

**Interviewer guide:**

Care home nursing healthcare provision

- What role to you have in relation to providing or supporting the heart failure healthcare of older patients living in a care home?
- To what extent do patients have their heart failure condition regularly assessed by care home nurses?
- Is there a particular profile of patient that requires higher levels of heart failure care in the care home?
- Other than care home nurses, what other people have a key role in the provision of heart failure care to these patients (e.g., family members, auxiliary, GP, other specialists).
- How much input does your care home nursing service have with colleagues from cardiology (e.g., cardiac nurses)?
- What sort of education or training is provided for care home nurses about heart failure healthcare of older patients and what are your thoughts on this? (e.g. is it mandatory training, does it meet your professional needs?)

Views and perceptions of heart failure healthcare service for older people living in care homes

- How important do you feel it is for care home nurses to be involved in the heart failure care of residents?
- What do you believe are the main barriers for care home nurses in providing heart failure healthcare to people living in care homes? (e.g. COVID-19, financial and time constraints, lack of equipment, lack of knowledge, safety, complex needs etc.)
- What helps you as a care home nurse to ensure optimum practice in relation to providing heart failure healthcare to your residents.
- How do you think we can improve provision of heart failure healthcare for older people living in care home settings?

*Conclusion:*

- Do you have anything else you would like to add that we haven’t already discussed?
- Thank you for your contribution.
- Ask the participant if there are any particular statements that they do not wish to be transcribed.

**Interviewer Gude During Constant Comparative Analysis**

- - How well did your pre-registration nursing program prepare you for managing heart failure in care home settings? What specific aspects of heart failure care do you feel were not adequately covered?
  - How do you manage the complexities of heart failure in residents with multiple comorbidities? What additional training do you think would be beneficial in this area?
  - Can you describe any recent continuing professional development opportunities you've had related to heart failure management? If none, what specific areas of heart failure care would you like to see covered in future CPD sessions?
  - How often do you have access to GP support for routine monitoring of heart failure patients? What challenges do you face in obtaining regular GP involvement?
  - Are you aware of any community-based heart failure specialist nurses or cardiologists that you can consult? If not, how do you think having access to such specialists would impact your ability to care for residents with heart failure?
  - What resources or tools do you currently use to stay updated on heart failure care guidelines? What additional resources do you think would be helpful?
  - When managing a resident with heart failure, who do you typically contact for external support? How do you decide when and whom to involve?
  - How do you approach discussions about advance care planning with families of residents with heart failure? What challenges do you face in these conversations?
  - How is information about residents with heart failure communicated between nurses and care assistants? What information do you think is most important to share?
  - How do you implement and manage exercise-based rehabilitation activities for residents with heart failure?
  - How confident do you feel in recognizing and managing heart failure symptoms in care home residents? What would help increase your confidence in this area?
  - Can you describe a situation where you felt well-supported in managing a resident's heart failure? What made this experience positive?
  - How do you handle situations where there's a misunderstanding or conflict with families about heart failure care? What strategies have you found effective?
  - Are there any interdisciplinary approaches or team meetings in your care home that include discussions about heart failure management? If so, how effective are these? If not, how do you think such approaches could be implemented
